# The association between timed up and go test and history of falls: The Tromsø study

**DOI:** 10.1186/1471-2318-7-1

**Published:** 2007-01-12

**Authors:** Gyrd Thrane, Ragnar M Joakimsen, Eline Thornquist

**Affiliations:** 1 Faculty of health sciences, Tromsø University College, N-9293 Tromsø, Norway; 2 Institute of clinical medicine, University of Tromsø, and Department of kidney diseases, University Hospital of North Norway, Tromsø, Norway; 3 Department of Nursing and Health Sciences, University of Tromsø, Tromsø, Norway

## Abstract

**Background:**

Fall-related injuries in older adults are a major health problem. Although the aetiology of falls is multifactorial, physical factors are assumed to contribute significantly. The "Timed up and go test" (TUG) is designed to measure basic mobility function. This report evaluates the association between TUG times and history of falls.

**Methods:**

A retrospective, observational, population-based study was conducted on 414 men and 560 women with mean age 77.5 (SD 2.3). TUG time and falls during the previous 12 months were recorded. Covariates were age, sex, medical history and health-related mobility problems. Means, confidence intervals and test characteristics for TUG were calculated. Odds ratios and influence of covariates were examined by logistic regression.

**Results:**

The mean TUG time was 11.1s (SD 2.5) among male non-fallers and 13.0s (SD 7.8) among fallers. The difference was 1.9s (95%CI 0.9–3.0). The odds ratio for fallers being in the upper quartile was 2.1 (95%CI 1.4–3.3). Adjusted for covariates, the odds ratio was (OR = 1.8, 95%CI 1.1–2.9). The corresponding mean was 13.0s (SD 5.74) among female non-fallers and 13.9s (SD 8.5) among fallers. The difference was 0.9 (95%CI -0.3–2.1). The odds ratio for fallers being in upper quartile was 1.0 (95%CI 0.7–1.4). The area under the ROC curve was 0.50 (95%CI 0.45–0.55) in women and 0.56 (95%CI 0.50–0.62) in men.

**Conclusion:**

TUG is statistically associated with a history of falls in men but not in women. The ability to classify fallers is poor, and the clinical value of the association is therefore limited.

## Background

Approximately 30% of adults over 65 years old fall each year [1,2]. The incidence is higher for persons 75 years or older [3]. The combination of a high incidence of falls and a high susceptibility to injury is a key concern in older adults [4]. To be effective, fall prevention programs have to include people at high risk [5]. Decline in physical mobility is probably a risk factor for falls [6-8], so assessment of physical mobility may be valuable for predicting falls and targeting prevention programmes.

The "Timed Up and Go test" (TUG) was presented in 1991 as a basic test for functional mobility [9]. The test measures speed during several functional manoeuvres, which include standing up, walking, turning and sitting down [9]. Limited training and equipment are required, and the test is therefore convenient in clinical settings. Good test-retest reliability (ICC = 0.97–0.99 and Spearmans = 0.93) [9-11] have been demonstrated in many studies, although a Canadian population-based study has questioned the test-retest reliability (ICC = 0.56)[12]. In the original article, TUG was proved to have good inter-rater reliability (ICC = 0.99) [9]. Other studies have supported this (ICC = 0.87–0.99) [10,13].

The theoretical validity of the test has not been evaluated. Neither is the test designed to assess all aspects of functional mobility or risk factors for falls. Therefore its validity has to rely on discriminative and predictive properties. The association with a history of falls has been evaluated previously; some studies suggest that TUG times differ between people with and without a history of falls [14-17]. These studies have been cited in different guidelines [4,18,19] for fall prevention and in textbooks of motor control in the elderly [20], and they may have had a substantial impact on general practice. Fall risks may be different across cultures and within different climates. The Tromsø Osteoporosis Study aims at preventing fall-related injuries in the Norwegian population. We therefore wanted to evaluate the association between TUG times and history of falls in a sample of older adults from this population.

## Methods

### Study population

**Figure 1:**
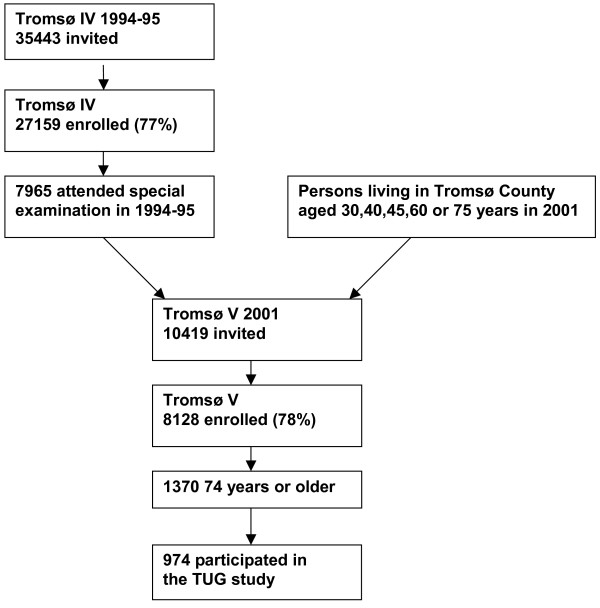
Study population.

Our study was part of the Tromsø Study, a longitudinal population-based multipurpose study focusing on lifestyle-related diseases. The first Tromsø study (Tromsø I) took place in 1974 and the fifth survey in 2001 (Tromsø V). Figure 1 shows the process for including participants. The invitations to Tromsø V were based on the subjects' participation in a special examination during the 1994 survey. All persons 55 to 74 years old, and 5–10% of persons 25–54 and 75–84 years old, were invited in 1994 and the response rate in was 76%. Persons still living in Tromsø in 2001 were invited to Tromsø V. Because of an ongoing national health survey, every person 30, 40, 45, 60 or 75 years old were also invited. Persons 74 years or older (n = 1370) met the criteria for participation in the TUG study. Three hundred and ninety-six subjects refused to participate or were excluded owing to logistic problems at the TUG station. Subjects were informed of the nature of the survey, and informed consent was obtained prior to the examination. The study was recommended by the Regional Committee of Medical Research Ethics and approved by the Norwegian Data Inspectorate.

### Measurements

TUG measurements were obtained using an ordinary armchair (47 cm high) and stopwatch. Subjects were seated with their back against the chair. They were instructed to stand up, walk three metres (to a mark on the floor), turn around, walk back to the chair and sit down. The task was to be done at an ordinary comfortable speed. The stopwatch was started on the word "go" and stopped as the subject sat down. The TUG time was measured in seconds (s). Normal TUG time is reportedly from 5.4s to 40.8 s, mean 15s (SD 6.5) [21]. Ten physicians, physiotherapists and research assistants performed the tests. Falls during the past year were recorded using a questionnaire and validated during an interview. The questionnaire was sent to the participants and was answered before the examination. During assessment, the interview was conducted prior to the TUG measurement. In the interview the examiner defined "fall" according to Lach et al. as "an unexpected loss of balance resulting in coming to rest on the floor, the ground, or an object below the knee level" [22]. There was a large discrepancy between the fall answers in the questionnaire and the interview. Because of this, and because questionnaires usually underestimate fall incidence [23], a person was defined as a faller if he/she had reported a fall in either the questionnaire or the interview, and a non-faller if the answer was no in both. The results presented in this paper are based on this definition of fall. For validation purposes, analyses were conducted with three other definitions of falls (fall according to questionnaire, interview or both).

Cofactors such as age, sex, medical history and health-related mobility problems were recorded in the questionnaire. Medical history included self-rated health and self-reported asthma, bronchitis, diabetes, osteoporosis, fibromyalgia, angina pectoris, myocardial infarction or stroke. Health-related mobility problems included problems with indoor mobility, outdoor mobility, social activities, using public transport and shopping. Because of a strong correlation between the health-related mobility variables, a new variable, number of health-related mobility problems, was created. This was an index variable counting the number of health-related mobility problems.

### Statistical analysis

The software package SPSS r11.0 (SPSS Inc. Chicago, Illinois) was used for analysis. Frequencies and proportions were calculated for categorical data, and means and standard deviations were calculated for normally distributed data. This was done on the data available for subjects invited to Tromsø V, for those who participated in the Tromsø V survey, and for subjects in the TUG study. The mean TUG results among fallers and non-fallers, mean differences, and 95% confidence intervals (95%CI) for difference were calculated. The odds ratio for being a faller in the upper quartile of the TUG results was computed by logistic regression. The area under the ROC curve and the confidence interval were calculated. Sensitivity, specificity, positive predictive value (PPV) and negative predictive (NPV) value were calculated for all cut-offs between 12 and 17 seconds.

Cross tables and chi-square were used to evaluate the relationship between covariates and falls. Covariates that were associated (p < 0.1) were included in the multivariate analysis. To control for all covariates, a backward conditional logistic regression was conducted on TUG times, age, medical history variables, and number of health-related mobility problems. All analyses were stratified by sex.

### Power analysis

If too many people were selected for the fall risk program, many persons not at risk of falling would be included and the intervention would be less effective. If the test can predict fallers, it is expected to do so at the upper percentiles. We chose an arbitrary cut-off of the upper 25 percentile, because it had to be selective for people at high risk of falls. With α = 0.05 and β = 0.20, and assuming that 25% of the population is exposed, this study has the power to detect gender-specific differences down to an odds ratio of approximately 1.9, which corresponds to a relative risk of 1.5. Our assessment is that TUG must have at least this predictive strength to be clinically relevant, so the study should be adequately powered.

## Results

### Study population

This study population totalled 974 persons, 414 (42.7%) men and 560 (57.3%) women (Fig. 1). The mean age was 77.5 (SD 2.3), range 74–89 years. According to the register of inhabitants in Tromsø 2001, our study included 34.4% of the age stratum 74–89. Most of the participants (98.1%) were between 74 and 81 years, which was 49.4% of this age stratum in the Tromsø population. The response rate was slightly higher in men than in women. The age- and sex-distributions of those who were invited to Tromsø V did not differ from the subjects in the TUG study sample. The distributions of medical history and health-related mobility variables did not differ between the subjects in the Tromsø V survey and the subjects in the TUG sample. Two percent were living in an institution. Three hundred and ninety-six persons, 230 (41.1%) women and 166 (40.1%) men, were defined as fallers. Table 1 summarizes the characteristics of the subjects. There was no age difference between fallers and non-fallers. The mean TUG was 11.8s (SD 5.4) among men and 13.3s (SD 7.1) among women.

### "Timed up and go" and falls

Table 2 summarizes the characteristics of fallers and non-fallers. The mean TUG was 11.1s (SD 2.5) among male non-fallers and 13.0s (SD 7.8) among fallers. The mean difference was 1.9s (95%CI 0.9–3.0). In females, the mean was 13.0s (SD 5.7) among non-fallers and 13.9s (SD 8.5) among fallers. The mean difference was 0.9 (95%CI 0.3–2.1). The changes in the means due to age adjustment were less than 0.05s. We therefore only present the crude estimates. The quartiles were 9, 11 and 14 s in men and 10, 12 and 15 s in women. The odds ratios for fallers being in the upper quartile were 2.1 (95%CI 1.4–3.3) in men and 1.0 (95%CI 0.7–1.4) in women. The area under the ROC curve was 0.50 (95%CI 0.45–0.55) in women and 0.56 (95%CI 0.50–0.62) in men. No specific cut-off point could be defined from the ROC analysis. Table 3 summarizes the TUG test characteristics.

### Confounders

Self-rated health, asthma, diabetes, osteoporosis, and all health-related mobility problems were associated with falls and were included in the multivariate analysis. Age, weight, bronchitis, fibromyalgia, angina pectoris, myocardial infarction and stroke were not associated and were not included in the model. The adjusted model in men included TUG (upper quartile) (OR = 1.8, 95%CI 1.1–2.9), asthma (OR = 2.5, 95%CI 1.1–5.5) and number of health-related mobility problems (OR = 1.3, 95%CI 1.1–1.7). In women, only health-related mobility problems (OR = 1.2 95%CI 1.0–1.3) was included in the adjusted model. Six hundred and sixty-eight persons (72.9%) answered the two fall questions consistently. The four different definitions of falls did not interfere with the main results of this study.

## Discussion

The main result of this study is that there is a relationship between TUG time and history of falls in men but not in women. Although the difference between male fallers and non-fallers is statistically significant, even after multiple adjustments it is relatively small, which weakens any clinical usefulness. This is confirmed by the poor ability to classify fallers and non-fallers.

### Selection bias

This study was supposed to reflect the general population, and one third of the total population in the age stratum 74–89 was included. This should make it represent the elderly Tromsø population, which should be representative of a white Caucasian elderly population, although our climate is extreme. The results are most representative for the age stratum 74–81 with about half the total population. Few of our subjects have problems with outdoor or indoor mobility and the results are not valid for frailer people. Only 2% of our subjects were living in an institution, compared to about 11% of the target population[24], and the results do not pertain to the institutionalized elderly.

### Information bias

As mentioned above, the reliability of the test has been documented in several studies [9-11,13]. TUG is not translated into Norwegian in a standardized way, and earlier reliability studies may not be compatible. This limits the validity of this study. However, we believe that the relatively simple protocol of the test reduces the bias from insufficient translation. Although the test is easy to perform, some experience is probably necessary for it to be reliable. The study is a large scale population study, similar to that of Rockwood et al. [12], who questioned the test-retest reliability of TUG. Lack of inter-tester reliability and test-retest examination is a drawback of this study.

There were inconsistent results from the fall questionnaire and interview, which is problematic. The reliability of fall questionnaires has been discussed by others [23], and the discrepancy between the two answers in this study confirms that there may be a recall bias. The proportion of falls depends on the definition of falls and the way in which fall data are collected. The analysis in this study was conducted with four different definitions of falls, and the different definitions did not alter the main results of our study. In any case, it is possible that other sampling methods, e.g. a fall calendar or interview with relatives, could have changed our results. Cognitive impairment may also increase the recall bias. Owing to the large number of participants we were not able to assess this. We can expect that 5–16% of the subjects suffered from mild cognitive impairment [25,26], and this may be a confounding factor in our material. Using more stringent or standardized methods for registering falls might give results different from ours. However, these are the fall-recording methods most likely to be used in clinical practice.

### Confounders

Validation of crude questionnaire data is scarce. However, our data are shown to be consistent by the high correlation between questions on mobility. There may be numerous other confounders that we have not measured, and these may explain the result we found for men. If TUG times were used as a test, however, one would probably not take confounders into account (i.e. one would use TUG as a marker of risk notwithstanding aetiology), so crude results may be most relevant to practice.

Age was expected to be an important confounder. The probable reason why this was not so is that most of the participants were aged between 74 and 81 years, so the results mostly represented this group. The differences between sexes could be attributed to the greater variance in TUG times among female non-fallers. Women also scored more poorly on all health-related mobility problems and self-rated health, which could explain the difference from the male population.

### Design

The aim of the study was to describe the predictive ability of TUG, and a prospective design would have been optimal. However, bias introduced by a retrospective design would be expected to make the association between fall risk and TUG times stronger. If a fall influenced mobility, it should decrease mobility and thereby increase the TUG time. Consequently, our results should overestimate the ability of TUG to predict fall risk.

The study has limited power; however, TUG time has to show a strong association with fall risk if it is to be useful as a predictive tool. This study is adequately powered to detect clinically relevant associations. Because more women than men were included, power is not the reason for the gender differences. The confidence intervals showed that a difference of 2.40 seconds between fallers and non-fallers would be significant. In a clinical setting, this difference would be very difficult to interpret owing to the variance of the TUG times in both groups. A larger study would not make this small difference clinically relevant, although it might find it statistically significant.

### Theoretical plausibility

Causes and predictors of falls may be due to individual, task and environmental factors [20]. Physical mobility is only one of these. In addition, problems of balance may result from sensory, motor or central problems. The task tested in TUG may not challenge to these systems sufficiently to detect problems that are important in a fall risk situation. More specific tests of strength, coordination, proprioception and vision may be more sensitive to these problems [3,6-8]. Falls also depend on the type of movement task performed while falling [1]. In daily life, people perform difficult tasks in a changing environment, with uneven or slippery surfaces, objects in our way and other dangers of displacing gravity. This requires effective sensory integration and perception of the situation. The highly standardized TUG test is very different from these and may be one of the reasons for the poor correlation with a history of falls.

### Consistency

The results of our study contradict the results of Gunter et al. [16], who found that TUG correctly classified 71.2% of the fallers and non-fallers in a group of community-dwelling older adults (p < 0.001). The mean age was 77.4 (SD 5.4) years, and 83% were women. The number of subjects was lower (156). In addition, the mean TUG times for fallers (8.91s) and non-fallers (7.54s) were different from ours, which points to differences in population or test procedures. Shumway-Cook et al. [14] found a sensitivity of 0.87 and a specificity of 0.87 with a cut-off 13.5 seconds. This study had a low number of participants (n = 30) and non-randomized selection, and there was a large difference in age and medical history between the fallers and non-fallers. We therefore believe our study to be more valuable for evaluating the association between TUG and history of falls in community-dwelling older adults. A prospective study from Taiwan has shown an association with falls (OR = 1.02, 1.01–1.03) but the area under the ROC curve was only 0.61 [27]. These results are very similar to ours. In another study, TUG time was a predictor of indoor falls in a population of Norwegian females [28]. Evaluation of test characteristics was not reported, and the clinical relevance is therefore difficult to compare and evaluate.

## Conclusion

There is a statistical association between TUG times and history of falls, but the clinical relevance of this association is limited. Even in a retrospective design, there is hardly any association between TUG and fall risk. Consequently, TUG may not be used as a test of fall risk in an ambulatory elderly population.

## Competing interests

The author(s) declare that they have no competing interests.

## Authors' contributions

GT carried out the analysis and interpretation and drafted the manuscript. RMJ was responsible for the design and data collection, and helped with the analysis and in drafting the manuscript. ET participated in the data analysis and interpretation and the preparation of the manuscript. All authors read and approved the final manuscript.

## Pre-publication history

The pre-publication history for this paper can be accessed here:



## Figures and Tables

**Table 1:** Characteristics of Subjects

	Females (Mean (sd)/n (%))	Males (Mean (sd)/n (%))
N	560	417
Age	77.45 (2.26)	77.44 (2.26)
Height (cm)	158.51 (6.10)	172.57 (6.60)
Weight (kg)	67.89 (12.07)	78.14 (12.51)
Self-rated health (good or very good)	224 (42.0)	217 (53.8)
Indoor mobility (no problems)	418 (85.8)	338 (90.9)
Outdoor mobility (no problems)	425 (88.8)	353 (95.9)
Social activities (no problems)	324 (75.0)	268 (80.7)
Public transport (no problems)	364 (81.4)	311 (91.2)
Shopping (no problems)	354 (75.0)	323 (90.7)

Sd = standard deviation; n = number of subjects. Proportions are based on number of subjects with valid answers.

**Table 2:** Characteristics of fallers and non-fallers

	Non fallers (Mean (sd)/n (%))	Fallers (Mean (sd)/n (%))
N	578	396
Age (years)	77,42 (2,24)	77,49 (2,29)
Sex (female)	330 (57,1)	230 (58,1)
Self-rated health (good or very good)	272 (61,8)	168 (38,2)
Number of health related mobility problems.	0,56 (1,16)	0,90 (1,52)
TUG (s)	12,15 (4,75)	13,48 (8,26)

**Table 3:** Test characteristics of TUG for identifying fallers

	**12s**	**13s**	**14s**	**15s**	**16s**	**17s**
**n (TUG>x)**	239/114	183/84	142/60	102/44	85/27	66/23
**Sensitivity**	0.44/0.37	0.35/0.30	0.26/0.20	0.20/0.18	0.17/0.11	0.14/0.11
**Specificity**	0.58/0.79	0.69/0.86	0.75/0.90	0.83/0.94	0.86/0.97	0.90/0.98
**PPV**	0.43/0.54	0.44/0.58	0.42/0.57	0.45/0.68	0.47/0.70	0.48/0.78
**NPV**	0.60/0.65	0.60/0.65	0.59/0.63	0.60/0.63	0.60/0.62	0.60/0.62

X – cut-off for TUG. n(TUG>x) – number of subjects above cut-off. PPV – positive predictive value. NPV – negative predictive value.

## References

[B1] Berg WP, Alessio HM, Mills EM, Tong C (1997). Circumstances and consequences of falls in independent community-dwelling older adults. Age Ageing.

[B2] Tinetti ME, Speechley M, Ginter SF (1988). Risk factors for falls among elderly persons living in the community. N Engl J Med.

[B3] Cesari M, Landi F, Torre S, Onder G, Lattanzio F, Bernabei R (2002). Prevalence and risk factors for falls in an older community-dwelling population. J Gerontol A Biol Sci Med Sci.

[B4] (2001). American Geriatrics Society; British Geriatrics Society; American Academy of Orthopaedic Surgeons Panel on Falls Prevention. Guideline for the prevention of falls in older persons.. J Am Geriatr Soc.

[B5] Gillespie LD, Gillespie WJ, Cumming R, Lamb SE, Rowe BH (2000). Interventions for preventing falls in the elderly. Cochrane Database Syst Rev.

[B6] Bueno-Cavanillas A, Padilla-Ruiz F, Jimenez-Moleon JJ, Peinado-Alonso CA, Galvez-Vargas R (2000). Risk factors in falls among the elderly according to extrinsic and intrinsic precipitating causes. Eur J Epidemiol.

[B7] Dargent-Molina P, Douchin MN, Cormier C, Meunier PJ, Breart G (2002). Use of clinical risk factors in elderly women with low bone mineral density to identify women at higher risk of hip fracture: The EPIDOS prospective study. Osteoporos Int.

[B8] Nevitt MC, Cummings SR, Kidd S, Black D (1989). Risk factors for recurrent nonsyncopal falls. A prospective study. JAMA.

[B9] Podsiadlo D, Richardson S (1991). The timed "Up & Go": a test of basic functional mobility for frail elderly persons. J Am Geriatr Soc.

[B10] Schoppen T, Boonstra A, Groothoff JW, de Vries J, Goeken LN, Eisma WH (1999). The Timed "up and go" test: reliability and validity in persons with unilateral lower limb amputation. Arch Phys Med Rehabil.

[B11] Steffen TM, Hacker TA, Mollinger L (2002). Age- and gender-related test performance in community-dwelling elderly people: Six-Minute Walk Test, Berg Balance Scale, Timed Up & Go Test, and gait speeds. Phys Ther.

[B12] Rockwood K, Awalt E, Carver D, MacKnight C (2000). Feasibility and measurement properties of the functional reach and the timed up and go tests in the Canadian study of health and aging. J Gerontol A Biol Sci Med Sci.

[B13] Morris S, Morris ME, Iansek R (2001). Reliability of measurements obtained with the Timed "Up & Go" test in people with Parkinson disease. Phys Ther.

[B14] Shumway-Cook A, Brauer S, Woollacott M (2000). Predicting the probability for falls in community-dwelling older adults using the Timed Up & Go Test. Phys Ther.

[B15] Salgado R, Lord SR, Packer J, Ehrlich F (1994). Factors associated with falling in elderly hospital patients. Gerontology.

[B16] Gunter KB, White KN, Hayes WC, Snow CM (2000). Functional mobility discriminates nonfallers from one-time and frequent fallers. J Gerontol A Biol Sci Med Sci.

[B17] O'Brien K, Culham E, Pickles B (1997). Balance and skeletal alignment in a group of elderly female fallers and nonfallers. J Gerontol A Biol Sci Med Sci.

[B18] NICE (2004). Clinical practice guideline for the assesment and prevention of falls in older people.

[B19] Close JC, Lord SL, Menz HB, Sherrington C (2005). What is the role of falls?. Best Pract Res Clin Rheumatol.

[B20] Shumway-Cook A, Wallacott MH (2001). Motor control Theory and Practical Applications.

[B21] Newton RA (1997). Balance screening of an inner city older adult population. Arch Phys Med Rehabil.

[B22] Lach HW, Reed AT, Arfken CL, Miller JP, Paige GD, Birge SJ, Peck WA (1991). Falls in the elderly: reliability of a classification system. J Am Geriatr Soc.

[B23] Cummings SR, Nevitt MC, Kidd S (1988). Forgetting falls. The limited accuracy of recall of falls in the elderly. J Am Geriatr Soc.

[B24] (2002). Helse, sosiale forhold og kriminalitet.

[B25] Hanninen T, Hallikainen M, Tuomainen S, Vanhanen M, Soininen H (2002). Prevalence of mild cognitive impairment: a population-based study in elderly subjects. Acta Neurol Scand.

[B26] Zanetti M, Ballabio C, Abbate C, Cutaia C, Vergani C, Bergamaschini L (2006). Mild cognitive impairment subtypes and vascular dementia in community-dwelling elderly people: a 3-year follow-up study. J Am Geriatr Soc.

[B27] Lin MR, Hwang HF, Hu MH, Wu HD, Wang YW, Huang FC (2004). Psychometric comparisons of the timed up and go, one-leg stand, functional reach, and Tinetti balance measures in community-dwelling older people. J Am Geriatr Soc.

[B28] Bergland A, Jarnlo GB, Laake K (2003). Predictors of falls in the elderly by location. Aging Clin Exp Res.

